# Hyperspectral and Thermal Imaging of Oilseed Rape (*Brassica napus*) Response to Fungal Species of the Genus *Alternaria*

**DOI:** 10.1371/journal.pone.0122913

**Published:** 2015-03-31

**Authors:** Piotr Baranowski, Malgorzata Jedryczka, Wojciech Mazurek, Danuta Babula-Skowronska, Anna Siedliska, Joanna Kaczmarek

**Affiliations:** 1 Institute of Agrophysics, Polish Academy of Sciences, Lublin, Poland; 2 Institute of Plant Genetics, Polish Academy of Sciences, Poznan, Poland; University of Nebraska-Lincoln, UNITED STATES

## Abstract

In this paper, thermal (8-13 µm) and hyperspectral imaging in visible and near infrared (VNIR) and short wavelength infrared (SWIR) ranges were used to elaborate a method of early detection of biotic stresses caused by fungal species belonging to the genus *Alternaria* that were host (*Alternaria alternata*, *Alternaria brassicae*, and *Alternaria brassicicola*) and non-host (*Alternaria dauci*) pathogens to oilseed rape (*Brassica napus* L.). The measurements of disease severity for chosen dates after inoculation were compared to temperature distributions on infected leaves and to averaged reflectance characteristics. Statistical analysis revealed that leaf temperature distributions on particular days after inoculation and respective spectral characteristics, especially in the SWIR range (1000-2500 nm), significantly differed for the leaves inoculated with *A*. *dauci* from the other species of *Alternaria* as well as from leaves of non-treated plants. The significant differences in leaf temperature of the studied *Alternaria* species were observed in various stages of infection development. The classification experiments were performed on the hyperspectral data of the leaf surfaces to distinguish days after inoculation and *Alternaria* species. The second-derivative transformation of the spectral data together with back-propagation neural networks (BNNs) appeared to be the best combination for classification of days after inoculation (prediction accuracy 90.5%) and *Alternaria* species (prediction accuracy 80.5%).

## Introduction

In natural, uncontrolled conditions, crops and other plants are exposed to a combination of various biotic and abiotic stresses that affect host metabolism and lead to large yield losses [1–4]. Biotic stresses are caused by living organisms, such as fungi, bacteria, viruses, and insects, which infect plants using various strategies. One of the most popular ways of pathogen invasion of plants is by penetrating stomata. The increase in water permeability of cell membranes is increased by producing specific compounds, which may affect cuticular and stomatal conductance [5,6]. After infection, plants activate their defence mechanisms and undergo alterations of growth and development. The most common plant response to infection is the accumulation of specific compounds promoting thermal and stomatal change, such as salicylic acid [5]. Chaerle and Van Der Straeten [7] reported that hydrogen peroxide produced by *Pseudomonas syringae* induced the closure of stomata.

It is expected that climate change also might have a significant influence on plant biodiversity as well as host-pathogen interactions [8]. Harvell et al. [9] suggested that climate warming can contribute to increasing pathogen development rate and sporulation, resulting in a greater number of generations per year. Moreover, milder winters relax restrictions on pathogen life cycles and modify susceptibility of host plants. Changes in these mechanisms may cause an increase in the number of invasive pathogens and enhance their pathogenicity.

During plant stress, absorption of incident light changes in both the visible and NIR ranges [10–12]. This is due to the decrease in leaf chlorophyll concentration and changes in other pigments [13–17]. The change of absorption consequently influences the reflectance of stressed plants, which can be visualised by hyperspectral imaging systems as locally changed spectral characteristics of leaf surfaces [18–21]. Delalieux et al. [22] used hyperspectral analysis to detect biotic stress in apple trees. These authors demonstrated that cell structural changes in the leaves, induced by the biotic stress, resulted in visible, chlorotic lesions that mostly impacted the reflectance values of the wavelengths in the range of 580–660 nm, which corresponds to regions of chlorophyll absorption. It confirms that infected plants have lower chlorophyll concentrations compared to those of non-infected plants. The multispectral and hyperspectral reflectance imaging systems also were used to study powdery mildew in wheat [23], tomato late blight [24], grey mould on eggplant leaves [25], and sunflower fields infested by *Ridolfia segetum* [26].

To evaluate the stage of infection by pathogens and physiological status of fruit tissue, machine learning methods were used and developed [27–29]. The changes in metabolic processes influencing stomatal closure mechanisms can be monitored by thermographic systems that show modifications in leaf temperature distributions [30,31].

Non-destructive methods, such thermography, successfully have been used to detect bacterial, viral, and fungal infections [32–36] and also to assess plant-pathogen interactions by monitoring patterns of leaf surface temperature [7,37]. Digital infrared thermography was successfully applied to find correlations between temperature and transpiration of cucumber leaves infected with *Pseudoperonospora cubensis* [36,38] and apple leaves infected by the apple scab fungus, *Venturia inaequalis* [39,40]. It was found that pathogen infections induced a decrease in leaf temperature 1–3 d before the appearance of visible symptoms. They also indicated the relationship between the percentage of diseased leaf area and the maximum temperature difference observed on the leaf by thermography.

One of the major plant pathogens is the fungus from the genus *Alternaria*. The genus *Alternaria* is ubiquitous and includes both plant-pathogenic and saprophytic species, which may affect crops in the field or cause harvest and postharvest decay of plant products. The taxonomy of the genus *Alternaria* recently has undergone great changes (it is not yet well-defined). In general, certain species of *Alternaria* infect only a well-defined range of host plants. However, under certain circumstances, pathogens can mutate and infect new plants. *Alternaria dauci*, which causes leaf blight of carrot, also can be pathogenic to rape [41]. Species belonging to *Alternaria* are able to produce numerous secondary metabolites, including phytotoxins, which play an important role in the process of pathogenesis [42]. Phytotoxin production depends on fungal species and character of individual strains as well as on environmental conditions. Generally, phytotoxins of *Alternaria* are strongly pathogenic to some plant species and are weakly or non-pathogenic to other plants [43].

Fungi of the genus *Alternaria* penetrate plant cells via stomata or directly through the cuticle and epidermis, but the frequency of stomatal penetration exceeds that of epidermal penetration [44]. The infestation of leaves of oilseed rape causes obstruction and dysfunction of stomata, which affects the physiological processes in plants. One such process is leaf transpirational water loss, which is determined by stomatal conductance [45]. When stomata are open, transpiration cools the leaf, but when stomata are closed, transpirational cooling is no longer possible. To properly use the knowledge of the actual temperature distribution on the leaf surface, it is important to be able to distinguish between natural variation (spatial and temporal) of the leaf temperature and its sensitivity to the studied process. Temperature of leaves is affected by a wide range of plant and environmental features; therefore, studies on the influence of biotic stresses should consider their dynamic changes. The best way to do this is to apply the leaf energy balance equation [6]. The studies of leaf radiation temperature in field and laboratory conditions were greatly facilitated by the use of non-contact infrared thermometers and thermographic systems.

The goal of this study was to elaborate the method of early detection of biotic stresses caused by fungi based on agrophysical measurements, such as temperature and reflectance, of plant tissues in visual and infrared ranges of wavelengths. The evaluation was performed for fungal species belonging to the genus *Alternaria* that were host and non-host pathogens to oilseed rape (*B*. *napus*).

## Materials and Methods

### Material and its preparation for measurements

Oilseed rape plants used in this study were grown at a 20/16°C day/night-temperature regime with a photoperiod of 14/10 h. Light and air conditioning was automatically controlled. The study was performed using a Polish variety of oilmid rape called Monolit, which was obtained using tissue culture techniques, resulting in small genetic diversity and high uniformity of plants. The seeds were sown in a 1:1 mixture of sterile peat:soil in 7 × 7-cm plastic pots in four replicates of 14 pots, each containing one plant. Plants were grown for 6 weeks, until the development of the third leaf. The inoculation was performed by spraying plants with spore suspensions, which were 1:1 mixtures of two isolates of different origins of each of the following species: *A*. *alternata*, *A*. *brassicae*, *A*. *brassicicola*, and *A*. *dauci* (Table 1). Leaves of control plants were sprinkled with water. The inoculum was obtained by growing the selected isolates of *Alternaria* on potato dextrose agar medium obtained by mixing 34 g of potato dextrose broth (Sigma, UK) and 20 g of technical agar (Sigma, UK) per 1 L of media.

**Table 1:** The origins of *Alternaria* species used in this experiment.

No.	Species	Isolate symbol	Plant	Location	Region	Season and Year
**1**	*A*.*alternata*	ATW 47-8-1	Oilseed rape	Wydartowo (51 °71’23”N 16 °80’4”E)	GPOL	Spring 2001
**2**		AWJ 46-3		Chlebowo (52 °2’23”N 14 °52’3”E)	OPOL	Autumn 2000
**3**	*A*. *brassicae*	ACC1		Cerekwica (51 °55’35”N, 17 °20’18”E)	GPOL	Spring 2004
**4**		Bor6		Borowo (52 °7’19”N 16 °47’10”E)	GPOL	Autumn 2002
**5**	*A*. *brassicicola*	ATW 42-2		Lasin (53 °31’15”N 19 °5’3”E)	KUJP	Spring 2001
**6**		ABCOLA		Baldy (53 °35’56”N 20 °36’24”E)	VMAZ	Summer 2010
**7**	*A*. *dauci*	ADAU1	Carrot	Baranowo (53 °10’34”N 21 °17’35”E)	GPOL	Autumn 2005
**8**		ADAU2		Torun (53 °1’1”N 18 °36’35”E)	KUJP	Autumn 2010

Explanations: GPOL, Great Poland (central-west Poland); KUJP, Kujavia-Pomerania (central); OPOL, Opole region (southwest); Varmia-Mazuria (northeast)

The isolates were grown on 90-mm Petri dishes filled with 20 mL of media for 14 d in darkness at 20°C. Spores were washed off the Petri plates using sterile, distilled water and adjusted to a concentration of 5 × 10^5^ of conidiospores per 1 mL. In the case of *A*. *brassicae* (a producer of very large spores), the concentration of spores was 5 fold lower (1 × 10^5^ spores per 1 mL of spore suspension). After the inoculation of plants, the relative air humidity was increased to 85% for 72 h by covering the plants with plastic hoods. The hoods were then removed, and high humidity (70%) in the glasshouse was maintained. Evaluation of disease severity was performed using a 10-grade scale (0–9), where 0 is a healthy plant and 9 is a plant completely damaged by the disease. The measurements were collected at 3, 7, 14, and 21 d after plant inoculation.

### Thermal imaging system

The SC620 thermographic camera (FLIR Systems, Inc., USA) was used, which is sensitive in the mid-wavelength infrared range (MWIR) of 8–13 μm. Using an uncoooled microbolometer with a format of 640 × 480 pixels, recording at 30 Hz in full resolution was possible. The thermal sensitivity expressed as the noise-equivalent temperature difference (NETD) of the system was 40 mK at an object temperature of 25°C. The spatial resolution of the camera was 0.65 mrad. Connection with a personal computer was possible via a firewire port with a transfer speed of 14-bit real-time video. A lens with an angular field of view of 24 × 18° was used. An integrated 3.2-megapixel visual camera for generating visual images was used to merge visual and thermal images to better identify selected parts of leaves during image analysis. All series of measurements were performed at 21°C air temperature and relative humidity of 70% in daylight. The distance between the camera lens and the leaf surface was 0.6 m. During the measurement of individual leaves, the thermal camera was positioned perpendicular to their surfaces at a distance of 1.5 m. The adopted emissivity coefficient of the leaves was 0.98.

### Hyperspectral imaging system

Two linear hyperspectral scanners were used as the hyperspectral system: a VNIR camera with an ImSpector V10E imaging spectrograph (400–1000 nm) and a SWIR camera with a N25E 2/3” imaging spectrometer (1000–2500 nm) (SPECIM, Finland). The cameras were mounted 40 cm above a conveyor belt that had the speed regulated for each camera (to perform line scanning of the leaves). The leaves from the pots selected for hyperspectral analysis were cut out directly before the measurements and put on the conveyor belt surface with their surfaces perpendicular to the camera axis. The illumination source in the system consisted of 12 20-W halogen lamps placed in the inside bottom part of a hemispherical diffuser. The diffuser allowed homogeneous illumination of the scanned surface of the rape leaves to be obtained. The measurements were performed in a dark room to prevent the influence of external illumination. For each camera, speed of the conveyor belt movement during line scanning was adjusted individually to suit differences in spatial resolution and integration time of the cameras. The average speed of the belt conveyor was 0.025 m/s. The resolution of the VNIR camera image was 1344 (spatial) by 1024 (spectral) pixels by 12 bits, which corresponded to a root mean square (rms) spot radius of less than 40 μm and spectral resolution of 6.8 nm (with 30-μm slit width). A lens with a focal length of 23 mm, F-number of 2.4, and maximum spatial image size of 14.4 mm was used with the VNIR camera.

The image from the SWIR camera had a resolution of 320 (spatial) by 256 (spectral) pixels by 14 bits, which corresponded to an rms spot radius of less than 15 μm and spectral resolution of 10 nm (30 μm slit). A lens with a focal length of 30.7 mm, F-number of 2.0, and maximum spatial image size of 12.8 mm was used with the SWIR camera. The exposure time for the VNIR camera was about 3.6 ms and for the SWIR camera about 7.2 ms. The lenses of VNIR and SWIR cameras were equipped with spectral flattening filters (SP-SFVNIR/40 and SP-SFSWIR/40 [SPECIM], respectively).

The hyperspectral images from the two cameras were recorded on a PC using SpectralDAQ 2.1 data acquisition software for SPECIM cameras. The acquisition time of one scan of the fruit surface for the VNIR and SWIR cameras was 5 s. For each series of measurements, white and dark calibrations were performed to obtain the reflectance *R* from the raw data.

### Analysing algorithms

#### Thermographic data analysis

The registered thermal images of oilseed rape were initially processed with ThermaCam Researcher Professional 2.9 software (FLIR Systems, USA). The areas of individual leaves were selected with the use of polygon selection tool. Temperatures of all pixels within each area were subsequently sent to the records of a common database, taking into account the day after inoculation and type of inoculant/control. Each variant of inoculant type/control and day after inoculation contained between 80 × 10^3^ and 200 × 10^3^ temperature values of all pixels from 20 leaves. The basic statistics of the leaf temperature distributions for each variant of the experiment were calculated with the use of RStudio integrated development environment for R (version 0.97.551).

#### Hyperspectral data analysis

Each hyperspectral image included a few (2–6) oilseed rape leaves recorded at the same time. From the hyperspectral cube of studied leaves, one image was selected to create the binary masks of all the leaves in the image (in the majority of cases, this was the 540-nm image). The binary masks of the leaf surfaces were put on the average image of these wavelengths to eliminate the background. The average reflectivity from all the pixels in each region of the leaf surface was calculated separately for all the bands. The average spectral characteristics of leaf areas of all studied plants were gathered in an Excel 2007 (Microsoft Corporation, USA) database, which was then transposed into Attribute-Relation File Format (ARFF), the native file format in the Waikato Environment for Knowledge Analysis [“Weka”].

The pre-treatment of spectral characteristics was completed with the use of The Unscrambler X 10.1 (CAMO Software, Norway). First, the raw spectral data were smoothed with a Gaussian filter. The second derivative was then calculated, with the Savitzky-Golay method (fourth-order polynomial and 11 smoothing points) applied. The second derivative is a measure of the change in the slope of the curve. It is not affected by any linear “tilt” that may exist in the data and is, therefore, a very effective method for removing both the baseline offset and slope from a spectrum.

Pre-processing of the hyperspectral data consisted of choosing, from the whole spectral range registered by the two cameras, the range in which the spectral characteristics of the signal were sufficiently strong. To avoid the low signal-noise ratio and diminish the problem of high dimensionality of feature spaces (known as the Hughes phenomenon), only the wavelengths ranging from 430–2376 nm were used for classification, with approximately 32-nm increments per pixel. This way, 61 channels represented independent variables in the created models.

The classification experiments were performed on the hyperspectral data of the leaf surfaces (251 instances were randomly selected for the training/testing set and 28 instances for the validation set). The experiment of learning and testing was repeated 10 times with random data selection (cross-validation method). For each fold, the proportion between the data used for learning and data used for testing was 10–90%. It has been confirmed [46] that the stratified 10-fold cross-validation is a standard evaluation technique in situations where only limited data is available, and it is regarded as the most rigorous. The idea of 10-fold cross-validation is that data are partitioned randomly into 10 complementary subsets. Each subset is held out in turn and the learning scheme trained on the remaining nine-tenths. Its error rate is then calculated on the holdout set. The learning procedure is executed a total of 10 times on different training sets.

All classification algorithms were implemented from the “Weka” [46]. In this study, two categories of “Weka” classifiers were used: functions and trees. Initially, the majority of available classifiers in these categories were tested on representative groups of training and testing data. Eight with the best prediction accuracies were chosen for comparison (these classifiers are presented in Table 2 together with a general description and the actual parameters determined in this study).

**Table 2:** Chosen features of the classifiers used in the study.

Type of classifier	Name of classifier’s library	Description of algorithm	Acronym	Chosen parameters of classifier
Bayes	Naive Bayes	Naive Bayes classifier that uses estimator classes. Numeric estimator precision values are chosen based on analysis of the training data.	NB	Use supervised discretisation: true; Debug: false;
				Use kernel estimator: false
Functions	Simple logistic	Builds linear logistic regression models with built-in attribute selection.	SL	Heuristic stop: 50; MaxBoostingIterations: 500; NumboostingIterations: 0
	LibSVM	A wrapper class for the libSVM tools. Allows users to experiment with one-class SVM, regressing SVM, and nu-SVM supported by LibSVM tool.	SVM	SVM Type: nu-SVC; Kernel type: linear; Ny: 0.5; Normalise: true;
				Probability estimates: true
	LibLINEAR	A wrapper class for the liblinear classifier.	LINE	SVM type: L2 loss support vector machines; Bias: 1.0;
				Normalise: true
	Multilayer perceptron	Uses back-propagation neural networks to classify instances.	BNN	AutoBuild: true; Learning rate: 0.3; Momentum: 0.2; Training time: 500
				Hidden layers = (attribs + classes) / 2
Trees	Functional trees	Classifier for building ‘functional trees’, which are classification trees that could have logistic regression functions at the inner nodes and/or leaves.	FT	BinSplit: false; Model type: FT; NumBoostingIterations:15
	Random forests	Classifier for constructing a forest of random trees.	RF	Debug: false;
				MaxDepth: 0;
				Num of features: 0;
				Num of trees: 10;
				Seed: 1
	J48	Classifier for generating a grafted (pruned or unpruned) C4.5 decision tree.	J48	Confidence factor: 0.25;
				Debug: false;
				The minimum number of instances per leaf: 2;
				Subtree raising: true

The Weka knowledge flow interface was used for all the studied classifiers in which two groups of dependent variables in classification models were used: days after inoculation (3, 7, 14, and 21 d after inoculation) and *Alternaria* species used for inoculation (*A*. *alternata*, *A*. *brassicae*, *A*. *brassicicola*, and *A*. *dauci*) as well as the control. This graphical interface allows the design and execution of configurations for streamlined data processing. The supervised classification models for the eight tested classifiers were created to distinguish between days after inoculation and between various *Alternaria* species. The parameters of the classifiers used to create the models are described in Table 2.

## Results

### Disease incidence and severity

**Fig 1:**
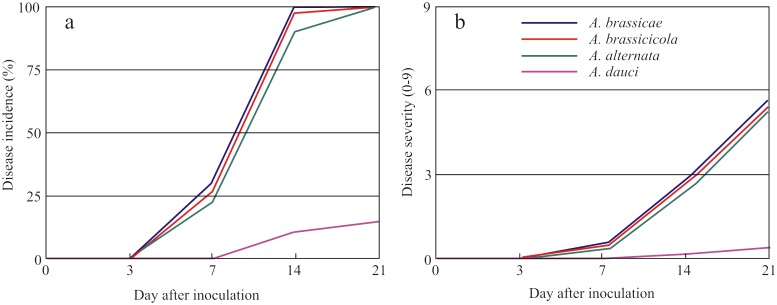
Disease incidence (a) and severity (b) of oilseed rape (*Brassica napus*) plants treated with four *Alternaria* species on various d after inoculation (dai). Disease incidence was calculated as the percentage of infected plants treated with *Alternaria* spp. Disease severity was measured according to a 0–9 scale, where 0 is a healthy plant and 9 is a plant completely damaged by the pathogen. *Alternaria* host species to oilseed rape: *A*. *brassicae*, *A*. *brassicicola*, *A*. *alternata*; non-host *Alternaria* species to oilseed rape: *A*. *dauci*.

The disease incidence greatly differed between *Alternaria* species and measurements in subsequent time points. No disease symptoms were observed on the third day after inoculation regardless of *Alternaria* species used for artificial infection. At 7 d after inoculation, three species for which oilseed rape is a host plant (*A*. *brassicae*, *A*. *brassicicola*, and *A*. *alternata*) caused small disease symptoms on approximately 25% of plants, ranging from 22.6% of plants infected by *A*. *alternata* to 30% of plants with disease symptoms caused by *A*. *brassicae* (Fig 1a). At this time point after inoculation, the mean disease severity on oilseed rape plants was about 0.5, which indicates small spots on leaves (Fig 1b). In addition, no disease symptoms were observed on oilseed rape leaves sprayed with the spores of *A*. *dauci*, which are pathogenic to carrot but not oilseed rape. Very small disease symptoms caused by these two species were visible for the first time 2 weeks post inoculation. At this time, *A*. *brassicae*, *A*. *brassicicola*, and *A*. *alternata* already showed considerable disease symptoms on 90.3–100% of plants. Three weeks after inoculation, all plants sprayed with the spores of these three species were infected, and the mean disease symptoms were in a narrow range—from 5.6 (*A*. *alternata*) to 5.9 (*A*. *brassicae*). Three weeks after inoculation, oilseed rape plants sprayed with the spores of *Alternaria* species to which they were host plants started to wilt and turn yellow. Disease patterns of *A*. *dauci* greatly differed from the other three studied species, although with time the disease symptoms were appearing on oilseed rape plants to a very small extent (mainly on the 21^st^ day after inoculation).

### Statistical analysis of temperature distribution of infected leaves

**Fig 2:**
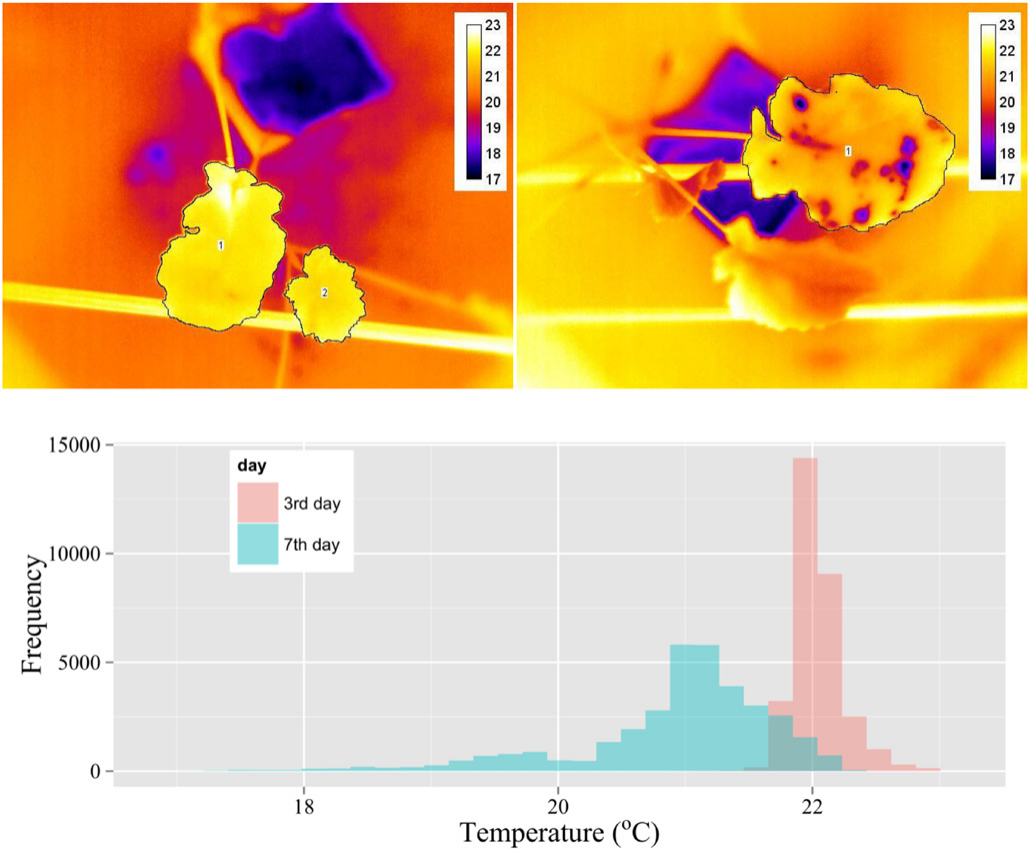
Thermal images of oilseed rape leaves inoculated with *Alternaria brassicae* after 3 (left) and 7 (right) d after inoculation (dai) with selected areas representing analysed leaf surfaces (temperature values in the legend in °C). Below, a histogram of temperature distribution of the leaves for these two days. Areas of the leaves used for histogram construction (two leaves for 3 dai and one leaf for 7 dai) are shown selected with polygon line.

The temperature distributions of infected and control leaves were analysed by selecting leaf areas in each thermal image with the use of the polygon area selection tool of the ThermaCAM Researcher software. Fig 2 shows exemplary images of oilseed rape leaves inoculated with *A*. *brassicae* 3 and 7 d after inoculation. On the third day after inoculation, the average leaf temperature was higher than on the seventh day, and the range of temperatures was much lower. The histogram obtained for the results of 7 d after inoculation shows an increased range of temperatures and a lower temperature median. The infected parts of leaves on day 7 are manifested as irregular areas of temperatures lower than the rest of the leaf.

**Fig 3:**
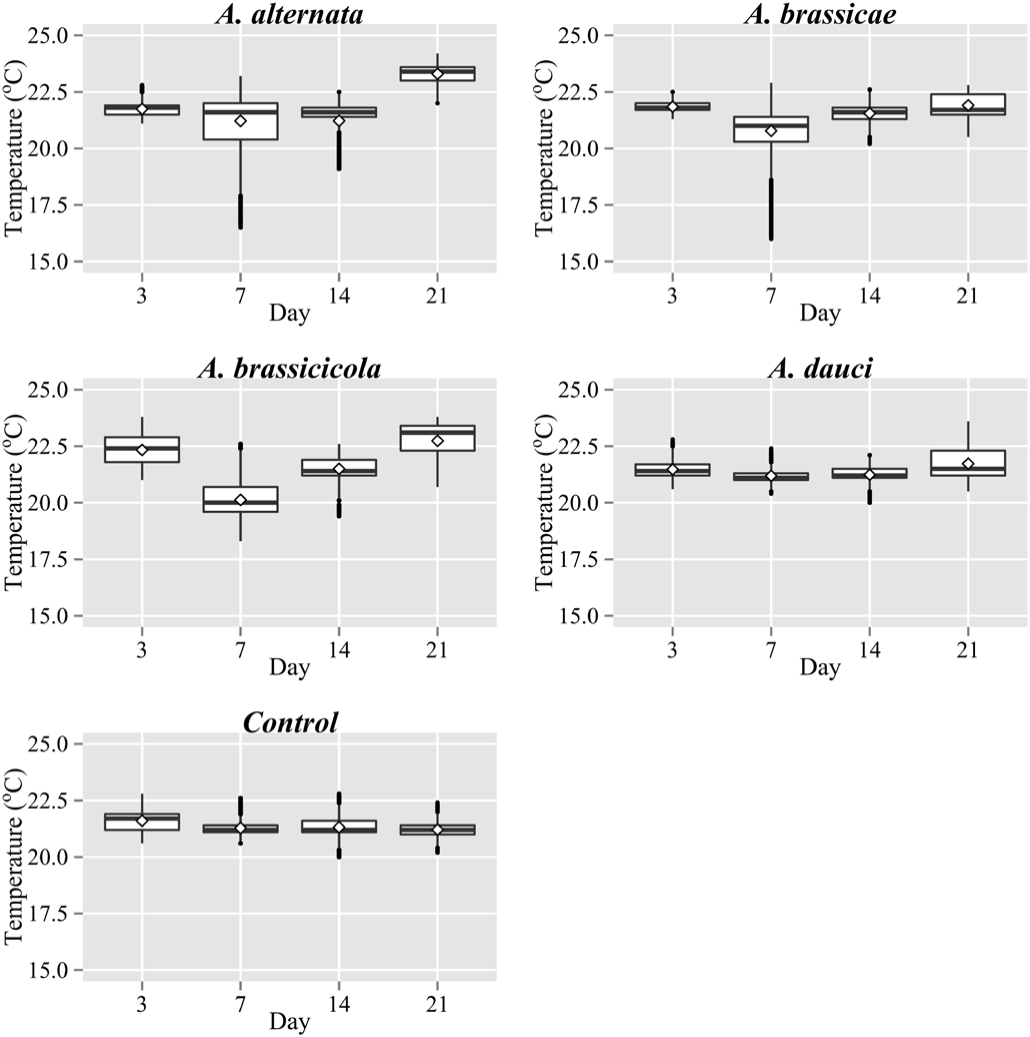
Box plots of temperature distributions during four studied periods after inoculation with various species of the genus *Alternaria* **.** The boxes in this plot range from the 25–75^th^ percentile of the data (inter-quartile range). The horizontal line within the box is the median (50^th^ percentile of the data). The diamond within the box is the mean value. The whiskers extend 1.5 times the inter-quartile range. The dots on the whiskers are the outliers.

The general statistics of the temperature distributions within the leaves infected with the four studied *Alternaria* species are presented as box plots (Fig 3). In each plot, the axis of abscissae represents days after inoculation. Each box plot was created taking into account the temperature values of all the pixels of 20 representative leaves of each category. Thus, each box refers to the statistical values of temperature of about 10^6^ points. The medians and means of all the infected leaves except the inoculation with *A*. *dauci* indicated a decrease of temperature during 3–7 d after inoculation and an increase during 7–21 d after inoculation. Regarding the spray with conidiospores of *A*. *dauci*, increases of mean temperature and median were observed only on 21 d after inoculation. It is also evident that the least variation of temperature within the studied period was observed for control plants, which were not infected with any species of *Alternaria*.

**Fig 4:**
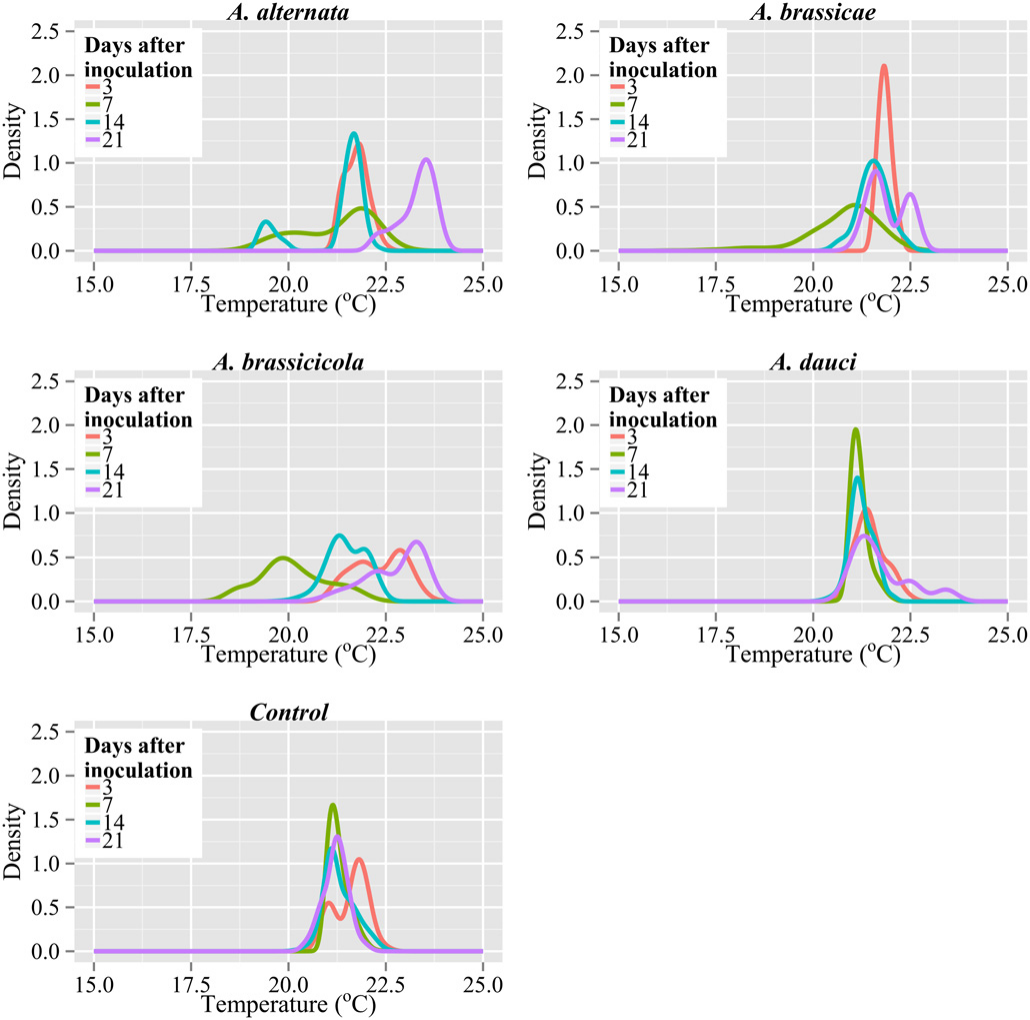
Density plots of leaf temperature distributions on succeeding days after inoculation for various types of inoculations **.** A kernel density curve is shown estimating population distribution based on the sample data. The adjust parameter, which is responsible for the amount of smoothing, was set to 4.

The distributions of leaf temperatures on particular days after inoculation for all the variants of the experiment were compared with the use of kernel density curves (Fig 4). A kernel density curve is an estimate of the population distribution based on the sample data. The vertical scale is calculated so the sum of the histogram bar areas equals unity. It was found that inoculation of oilseed rape leaves with all *Alternaria* species caused changes in temperature distributions on particular days after inoculation compared to control leaves (Fig 4). In the majority of cases, the distributions of infected leaves strongly differed from a normal distribution. The density peaks for the studied days after inoculation indicated high differentiation, especially for *A*. *alternata*, *A*. *brassicae*, and *A*. *brassicicola*. The density curves of *A*. *dauci* were very similar to the respective control density curves (especially for 7 and 14 d after inoculation). In control plants, the temperature distributions were very similar throughout the experiment.

**Fig 5:**
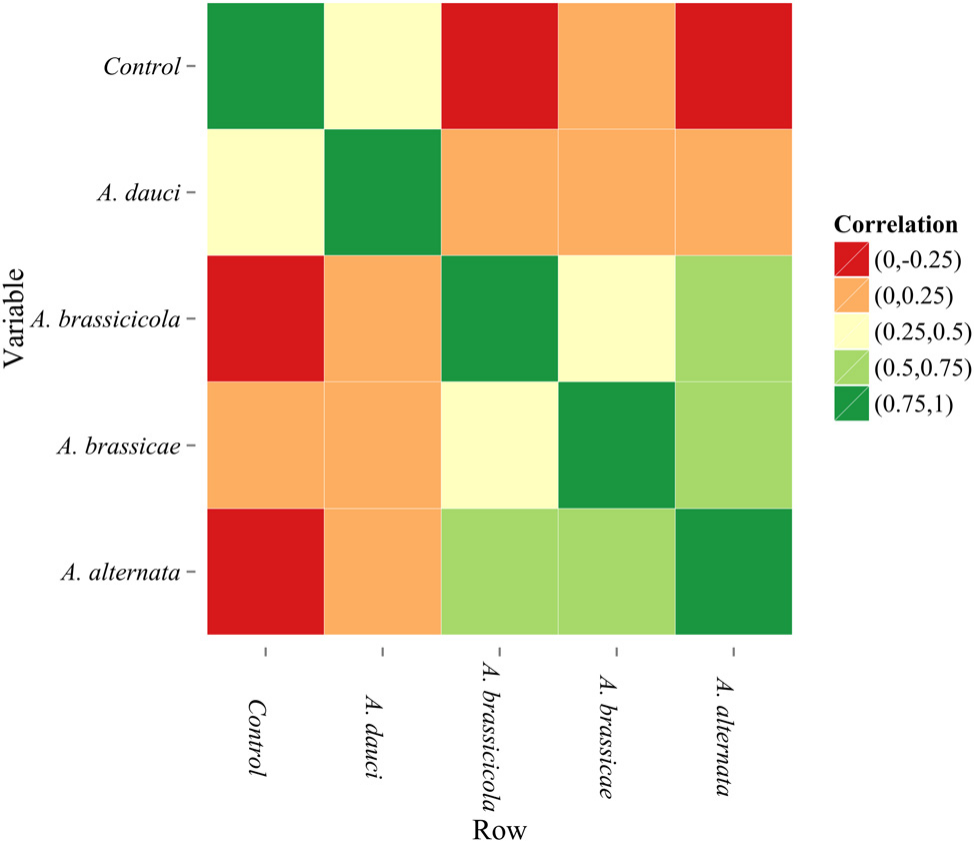
Correlation plot for control leaves and various types of inoculation. The plot is a visualisation of the strength of correlations among leaf temperatures in various types of the experiment. The legend divides correlation coefficients into five ranges.

To estimate a rank-based measure of association between leaf temperature distributions for control plants and those infected with various *Alternaria* species, a correlation plot was created (Fig 5). In this plot, the correlation coefficients were divided into five ranges. It was demonstrated that the temperature of control leaves and those infected with *A*. *dauci* had low correlations with other variants of the experiments. A considerably high correlation existed between leaf temperature and other variants (e.g., the temperature of leaves infected with *A*. *alternata* was strongly related to the temperature of leaves infected with *A*. *brassicicola* and *A*. *brassicae* species). The infection with *A*. *dauci* resulted in leaf temperature distributions that were significantly different from those of leaves infected with other *Alternaria* species.

### Reflectance spectra

**Fig 6:**
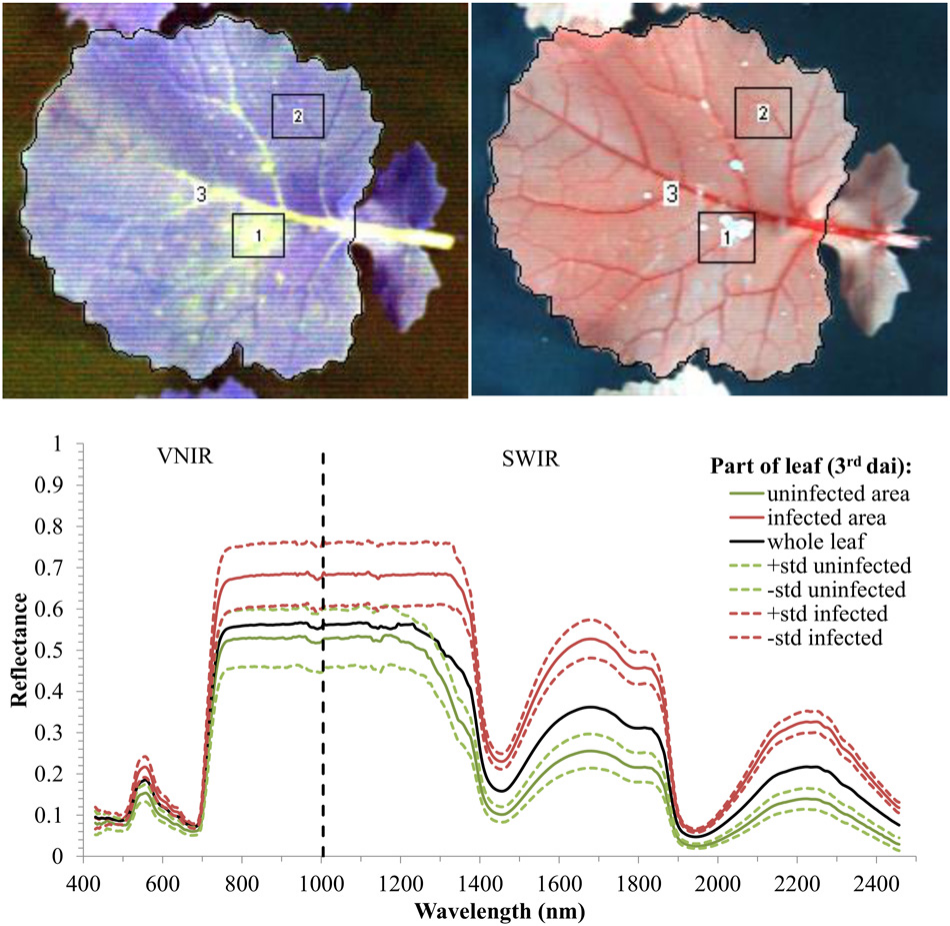
False-colour compositions of visible and near infrared (VNIR) (503.5 nm, 660.1 nm, and 970.5 nm) and short wavelength infrared (SWIR) (1200.3 nm, 1759.2 nm, and 2202.3 nm) hyperspectral bands of an oilseed rape leaf inoculated with *Alternaria alternata* **.** The lower plot shows spectral characteristics within selected regions of the image representing the infected area, uninfected area, and entire leaf with standard deviation (std).

Spectral characteristics of the leaves indicated high differentiation of their reflectance response in the areas with the symptoms of *Alternaria* infection and in the uninfected areas. In Fig 6, exemplary false-colour images of oilseed rape leaves 3 d after inoculation in VNIR (left) and SWIR (right) ranges are presented together with reflectance spectra in the selected areas. These three regions correspond to: 1, the part of the leaf with visible symptoms of infection; 2, the part of the leaf without any symptoms of infection (uninfected area); and 3, the area of the entire leaf. Additionally, the standard deviation lines for uninfected and infected regions are included. The reflectance in the infected area was higher than in the uninfected area in the entire VNIR-SWIR range, and non-overlapping standard deviation lines indicate good separation for the areas of the leaf with and without symptoms of the infection. In the visible range of the spectrum, the absolute mean difference values of the reflectance between areas with and without disease symptoms were highest from 545–700-nm wavelengths (which include the chlorophyll absorption red-edge sub-region). The best separation between the uninfected and infected areas is observed in the SWIR range and also in the water-absorption bands (1470 and 1900 nm).

**Fig 7:**
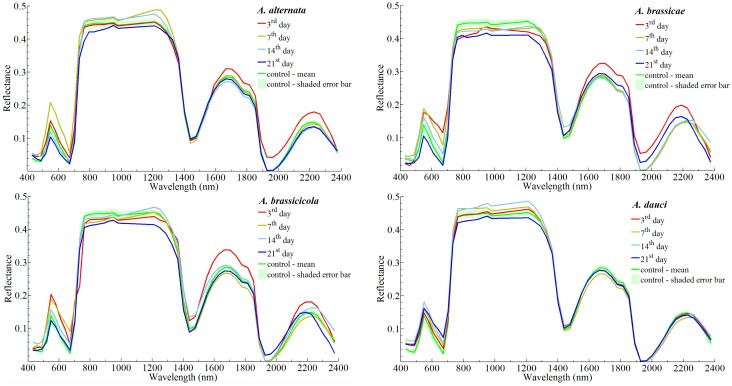
Spectral characteristics of rape leaves infected with four species of *Alternaria* fungi during four studied periods.

A comparison of the mean reflectance spectra of the oilseed rape leaves on various days after inoculation (3–21 d) by *Alternaria* species and controls (uninfected leaves) is presented in Fig 7. In these plots created separately for all the studied *Alternaria* species, the mean spectral characteristic of the control variant taken from four studied days after inoculation is added together with the shade of the error bar, representing standard deviations. Considerable differences of the spectral characteristics in the entire studied range (400–2400 nm) were observed between particular days after inoculation (especially for *A*. *alternata*, *A*. *brassicae*, and *A*. *brassicicola*). In the range of 740–1250 nm there are significant differences in spectral characteristics of the studied variants of the experiments. For *A*. *brassicae*, and *A*. *brassicicola* the spectral characteristics obtained for particular days after inoculation are in majority of cases lower than control. Moreover, in the range of 1400–2400 nm, the leaves inoculated with *A*. *dauci* show different spectral profiles than the other variants. This variant of the experiment show very low variation of reflectance between particular days after inoculation in this spectral range. In case of other three variants (*A*. *alternata*, *A*. *brassicae*, and *A*. *brassicicola* infected leaves) of the experiment there are noticeable decreases of reflectance after 3rd day after inoculation.

These results confirm different development and symptoms of the inoculation by *A*. *dauci* on rape leaves than those from infection with other studied *Alternaria* species.

### Supervised classification models

The final results of classification models for distinguishing days after inoculation are presented in Table 3. For the training/test data set, the best prediction accuracies were obtained for the BNN (90.5% of correctly classified instances), FT (86.7%), and SL (86.3%) ones. The analysis of the models using the validation set resulted in the best accuracy with the BNN model (93.3% of correctly classified instances), and it is higher than the other models. The lowest prediction accuracy was obtained for the RF models, with 75.1% of correctly classified instances.

**Table 3:** Results of classification models for distinguishing day after inoculation.

Classification model for distinguishing day after inoculation	Training/test set	Validation set				
	Correctly classified instances (%)	Kappa statistic	Root mean squared error	Correctly classified instances (%)	Kappa statistic	Root mean squared error
NB	80.4	0.75	0.30	79.5	0.72	0.14
SL	86.3	0.79	0.20	83.2	0.76	0.23
SVM	83.1	0.76	0.22	79.7	0.72	0.22
LINE	80.5	0.73	0.30	82.5	0.76	0.28
BNN	90.5	0.86	0.18	93.3	0.89	0.15
FT	86.7	0.83	0.20	82.6	0.76	0.25
RF	82.6	0.77	0.24	75.1	0.67	0.25
J48	85.8	0.81	0.26	78.9	0.72	0.32

**Fig 8:**
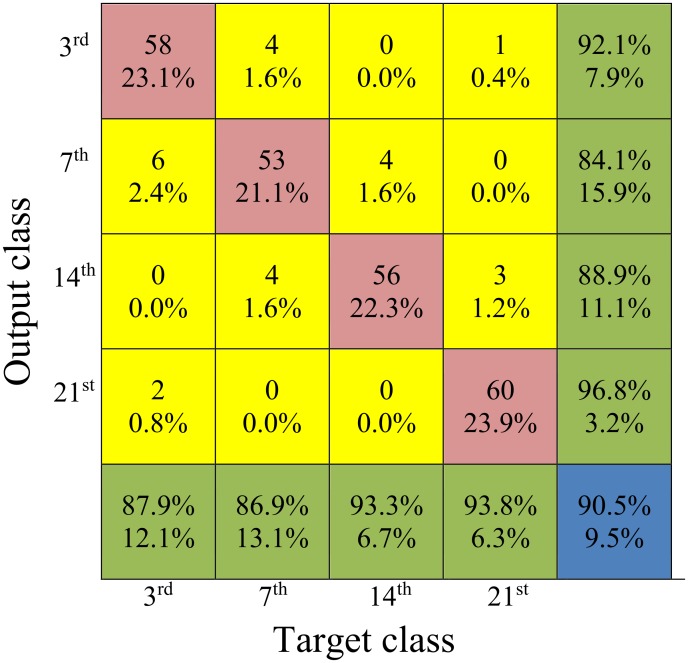
Confusion matrix obtained with 10-fold cross-validation for the back-propagation neural network model for the studied days after inoculation (dai). The upper number in each entry of the matrix is the average number of actual recognised classes in testing mode; the bottom one (its percentage) refers to the total number of cases used in testing (251). The diagonal entries of the matrix represent the mean quantities of the properly recognised cases (the upper value) and also their ratios with respect to the total representation of all testing data (the lower values are expressed as a percentage). Each entry outside the diagonal indicates an error (the number of misclassifications and its relative value). The last column of the matrix represents the total percentage measure of accuracy of actual recognition for the class indicated by the classifier. The upper number in this column represents the ratio of the number of the properly recognised cases to the total number of cases indicated by the particular output. The bottom numbers in the last column represent false alarm ratios. The last row of the matrix represents the ratios of the number of properly recognised cases to the total number of true cases (targets). The bottom numbers in this row are the misclassification ratios (the complement of sensitivity to one).

To illustrate how the cases belonging to different classes were classified by the particular models, confusion matrices were created. An example of a confusion matrix for the BNN is presented in Fig 8. The rows in the matrix represent the actual outputs, while the columns represent the targets. The highest number of misclassifications occurred 7 d after inoculation (10 cases), whereas the lowest number of misclassified cases occurred 21 d after inoculation (two cases). At 7 d after inoculation, the total percentage measure of accuracy of actual recognition (84.1%) was lower than that of the other classes. The highest percent of correctly classified cases was observed 21 d after inoculation (96.8%). The lowest misclassification ratio occurred 21 d after inoculation. These results confirm the fact that with the development of leaf infection, symptoms are better recognised in reflectance spectra.

The general information on prediction accuracy of the eight created models for distinguishing species of *Alternaria* is presented in Table 4. In this group of models, the best prediction accuracies for the training test/data set and also for the validation set were observed for BNN models (80.5% of correctly classified instances for training and 82.3 for validation sets). The other models had considerably lower accuracies (the percentage of correctly classified instances ranged from 49.8–59.3% for the training/testing set and 53.6–68.5% for the validation set).

**Table 4:** Results of classification models for distinguishing species of *Alternaria*.

Classification model for distinguishing species of Alternaria	Training/test set	Validation set				
	Correctly classified instances (%)	Kappa statistic	Root mean squared error	Correctly classified instances (%)	Kappa statistic	Root mean squared error
NB	49.8	0.38	0.33	53.9	0.44	0.33
SL	54.4	0.46	0.30	53.6	0.43	0.32
SVM	52.6	0.44	0.32	57.6	0.46	0.33
LINE	53.1	0.45	0.37	53.9	0.42	0.36
BNN	80.5	0.69	0.24	82.3	0.75	0.21
FT	59.3	0.50	0.31	58.7	0.47	0.31
RF	55.2	0.46	0.29	68.5	0.65	0.27
J48	53.4	0.44	0.35	54.3	0.43	0.34

**Fig 9:**
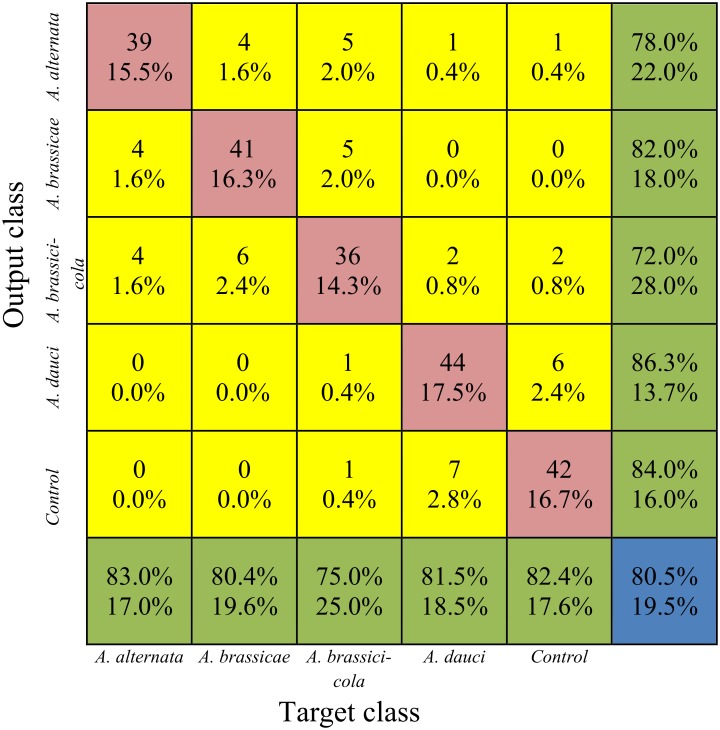
The confusion matrix obtained with 10-fold cross-validation for the back-propagation neural network model for the studied *Alternaria* species. The parameters of the matrix are analogous to those in Fig 8.

The performance of the BNN classification model that exhibited the highest prediction accuracy among the studied models of detecting species of *Alternaria* is presented in Fig 9. The lowest number of misclassifications (seven cases) and the highest prediction accuracy (86.3%) were obtained for *A*. *dauci*. The second highest prediction accuracy was obtained for the control leaves (84.0%), with only eight misclassified cases. The other *Alternaria* species exhibited lower prediction accuracies (72–82%).

## Discussion

It has been confirmed in this study that thermography and hyperspectral imaging technologies have large potential for studying infection of oilseed rape plants with *Alternaria* species. Analysis of radiation temperatures and reflectance spectra of infected leaves revealed considerable changes in time (3 weeks after inoculation) of these characteristics. For *Alternaria* species that are host to oilseed rape, a characteristic decrease in temperature occurred between 3 and 7 d after inoculation as well as an increase in temperature in further periods of the experiment. Similar leaf temperature characteristics were obtained for the infection of wheat plants with powdery mildew [47]. The initial leaf surface cooling was caused by growing mycelia, which have a very low evaporation resistance (increase of transpiration observed). During later infection stages (7–21 d after inoculation), the affected leaf parts were damaged, the tissue dried, and the temperature increased.

The results of supervised classification confirm that with the development of leaf infection, symptoms are better recognised in reflectance spectra. Similar influences of pathogens on leaf reflectance as a function of developmental stage of disease were obtained for sugar beet leaves infected by Cercospora leaf spot, powdery mildew, and leaf rust [11]. Both thermographic and hyperspectral measurements indicated different mechanisms of development and physiological features of oilseed rape inoculation with *A*. *dauci*. The leaf temperature changes on particular days after inoculation and spectral characteristics, especially in the SWIR range for inoculation by *A*. *dauci*, significantly differed from the other species of *Alternaria* as well as leaves of non-treated plants. These results confirm that symptoms of *A*. *dauci* infection on leaf surfaces differ from those of the other *Alternaria* species infections [41,42].

The thorough comparison of several pre-processing procedures and classifiers in these experiments, including four species of *Alternaria* and four periods after inoculation, indicated that second-derivative transformation of the spectral data, in conjunction with back-propagation neural networks, is the best combination for classification of days after inoculation and *Alternaria* species. The results indicating that reflectance in the areas infected by *Alternaria* species was higher compared to that of uninfected areas for the water absorption bands suggest that development of leaf infection and desiccation of the leaf tissue processes are in agreement with earlier studies [48–51].

## Conclusions

These results revealed good applicability of thermography and hyperspectral imaging in the VNIR and SWIR regions for studying the development of *Alternaria* infection in leaves of oilseed rape within a 3-week period after inoculation. Implementation of the elaborated algorithms of the supervised classification of detecting *Alternaria* species and the time after inoculation will require additional multisensory studies on interactions between physiological processes within the affected tissues and their thermal responses (possibly with the use of active thermography) and reflectance spectral characteristics. The knowledge of understanding the response of infected leaves in various ranges of the electromagnetic spectrum require inclusion of the entire range of physiological and genetic data.
